# Deep learning estimation of northern hemisphere soil freeze-thaw dynamics using satellite multi-frequency microwave brightness temperature observations

**DOI:** 10.3389/fdata.2023.1243559

**Published:** 2023-11-17

**Authors:** Kellen Donahue, John S. Kimball, Jinyang Du, Fredrick Bunt, Andreas Colliander, Mahta Moghaddam, Jesse Johnson, Youngwook Kim, Michael A. Rawlins

**Affiliations:** ^1^Numerical Terradynamic Simulation Group, University of Montana, Missoula, MT, United States; ^2^Department of Computer Science, University of Montana, Missoula, MT, United States; ^3^Jet Propulsion Laboratory, California Institute of Technology, Pasadena, CA, United States; ^4^Ming Hsieh Department of Electrical and Computer Engineering, University of Southern California, Los Angeles, CA, United States; ^5^Department of Biology, College of Science, United Arab Emirates University, Al Ain, United Arab Emirates; ^6^Department of Earth, Geographic, and Climate Sciences, University of Massachusetts, Amherst, MA, United States

**Keywords:** soil freeze-thaw, microwave, SMAP, AMSR, machine learning, neural network

## Abstract

Satellite microwave sensors are well suited for monitoring landscape freeze-thaw (FT) transitions owing to the strong brightness temperature (TB) or backscatter response to changes in liquid water abundance between predominantly frozen and thawed conditions. The FT retrieval is also a sensitive climate indicator with strong biophysical importance. However, retrieval algorithms can have difficulty distinguishing the FT status of soils from that of overlying features such as snow and vegetation, while variable land conditions can also degrade performance. Here, we applied a deep learning model using a multilayer convolutional neural network driven by AMSR2 and SMAP TB records, and trained on surface (~0–5 cm depth) soil temperature FT observations. Soil FT states were classified for the local morning (6 a.m.) and evening (6 p.m.) conditions corresponding to SMAP descending and ascending orbital overpasses, mapped to a 9 km polar grid spanning a five-year (2016–2020) record and Northern Hemisphere domain. Continuous variable estimates of the probability of frozen or thawed conditions were derived using a model cost function optimized against FT observational training data. Model results derived using combined multi-frequency (1.4, 18.7, 36.5 GHz) TBs produced the highest soil FT accuracy over other models derived using only single sensor or single frequency TB inputs. Moreover, SMAP L-band (1.4 GHz) TBs provided enhanced soil FT information and performance gain over model results derived using only AMSR2 TB inputs. The resulting soil FT classification showed favorable and consistent performance against soil FT observations from ERA5 reanalysis (mean percent accuracy, MPA: 92.7%) and *in situ* weather stations (MPA: 91.0%). The soil FT accuracy was generally consistent between morning and afternoon predictions and across different land covers and seasons. The model also showed better FT accuracy than ERA5 against regional weather station measurements (91.0% vs. 86.1% MPA). However, model confidence was lower in complex terrain where FT spatial heterogeneity was likely beneath the effective model grain size. Our results provide a high level of precision in mapping soil FT dynamics to improve understanding of complex seasonal transitions and their influence on ecological processes and climate feedbacks, with the potential to inform Earth system model predictions.

## 1 Introduction

Landscape freeze-thaw (FT) retrievals from satellite microwave remote sensing are well established as an effective proxy of frozen temperature controls on the seasonality of hydro-ecological processes (Zhang et al., 2004; McDonald and Kimball, 2006; Parazoo et al., 2018). More than half of the global land area is affected by seasonal freezing and thawing that can profoundly affect surface energy budgets and hydrologic activity, vegetation growth, and ecological trace gas dynamics (Kim et al., 2017). Microwave sensors are uniquely capable of detecting and monitoring FT transitions due to the strong microwave permittivity response to changes in landscape liquid water abundance between predominantly frozen and thawed states. The lower frequency (i.e., ~ ≤Ku-band) microwave measurements available from many operational polar-orbiting satellites are also insensitive to solar illumination and atmospheric contamination, enabling consistent observations in nearly all weather conditions.

Relatively long-term FT environmental data records have been developed by exploiting similar overlapping satellite microwave brightness temperature (TB) retrievals from the Scanning Multichannel Microwave Radiometer (SMMR), Special Sensor Microwave Imager (SSM/I), and Advanced Microwave Scanning Radiometers (AMSR-E/2) (Jin et al., 2009; Kim et al., 2017; Hu et al., 2019). These records exploit higher frequency (Ka-band) microwave channels that are more sensitive to the surface skin-layer, whereas the NASA Soil Moisture Active Passive (SMAP) (Entekhabi et al., 2010) and ESA Soil Moisture and Ocean Salinity (SMOS) (Mecklenburg et al., 2016) satellite Earth missions produce operational FT classification records from lower frequency (L-band) TB retrievals that have greater characteristic sensitivity to soil (~0–5 cm depth) conditions under low to moderate vegetation cover (Rautiainen et al., 2016; Derksen et al., 2017; Roy et al., 2020).

FT classification algorithms have generally exploited the magnitude of the observed TB difference from a FT reference state to derive a discrete, categorical classification of the predominant frozen or non-frozen condition at each time step (Kim et al., 2017); however, the simple binary nature of the retrieval limits the potential information available and the utility of the data for some applications (Bateni et al., 2013; Farhadi et al., 2015). The sensitivity of FT retrievals to different landscape elements is affected by the sensor frequency and polarization, with lower frequency channels having larger characteristic sensitivity to deeper vegetation, snow, and soil layers (Prince et al., 2018). However, the classification algorithms and retrievals have had only limited success in distinguishing soil conditions from the aggregate landscape FT signal (Bateni et al., 2013; Podest et al., 2014; Chen et al., 2019; Hu et al., 2019). Most operational satellite FT products were developed from single sensor observations (e.g. AMSR, SMAP; Derksen et al., 2017; Kim et al., 2017) and represent aggregate landscape FT conditions without distinguishing the FT states of land components such as snow, vegetation, and soil (Du et al., 2019; Mavrovic et al., 2023). The satellite FT products generally met mission requirements for global accuracy, but showed greater uncertainty during seasonal transitions, winter, and complex landscape conditions (Kim et al., 2019). The lack of differentiation and precision can limit capabilities for more effective monitoring and better understanding of the complexity of the FT transition and its biophysical linkages (Colliander et al., 2012; Roy et al., 2020).

More sophisticated methods have been developed blending multi-source information to enhance FT spatial resolution and accuracy (Johnston et al., 2021; Zhong et al., 2022), to better distinguish soil FT processes (Gao et al., 2018; Chen et al., 2019), and to obtain more continuous and probabilistic FT retrievals (Zwieback et al., 2012; Walker et al., 2022). Empirical machine learning methods have shown particular promise for FT classification (Li et al., 2022; Zhong et al., 2022). The resulting models can provide an efficient means for blending multi-scale and multi-source data as key predictors, while producing favorable accuracy without the need for a priori assumptions about the driving variables or physical processes involved. Unlike physical retrieval algorithms and process model approaches, machine-learning methods are also able to provide a robust best-fit estimation of FT status with minimal bias. However, the resulting model predictions may only be valid within the domain and range of conditions defined by the available ground truth observations used for model training. In addition, the machine learning approaches currently used in FT classifications are mainly derived from conventional shallow learning methods (e.g., Random Forest; Li et al., 2022; Zhong et al., 2022). Compared with more sophisticated deep learning (DL) methods, the shallow learning models typically have simple layered representations of the data and rely on manually designed features; whereas, DL models are characterized by successive layered data representations and automatic feature extraction from the multi-dimensional images (Schmidhuber, 2015). The DL methods have shown promise in uncovering subtle or hidden patterns in multi-source geospatial data sets (DeLancey et al., 2019), but have seen limited application in addressing satellite FT classification problems.

In this study, we applied a DL framework to develop a continuous daily record of near-surface soil FT conditions over the Northern Hemisphere (NH) and detailed the procedures of data sampling, preparation, projection, and fusion for training and validating the DL network. The DL method is a form of machine learning employing a multi-layer convolutional neural network for multi-level feature extraction and FT pattern recognition using satellite observations and other geospatial data encompassing NH land areas. The DL method was driven by satellite multi-frequency TB observations from SMAP and AMSR2, and trained using daily soil temperatures from sparse *in situ* weather station measurements and gridded meteorological data from global reanalysis. The resulting soil FT classification record is mapped to a 9 km resolution NH polar grid consistent with the SMAP TB inputs, and encompasses a five-year period (2016–2020) defined from available overlapping SMAP and AMSR2 annual records. Unlike other more established FT records that define a discrete binary landscape FT classification, the DL soil FT classification provides an additional continuous variable estimate of the probability of frozen soil conditions for each grid cell. The primary objectives of this study were to: (1) clarify the potential added value to the DL data model of the SMAP L-band (1.41 GHz) TB observations in determining soil FT conditions relative to the higher frequency (18.7, 36.5 GHz) AMSR2 TB inputs; and (2) assess the general performance and accuracy of the estimated soil FT classification. The following sections describe the data and DL method used for the soil FT predictions, the resulting product performance and validation assessment, and a summary of the key results and their significance.

## 2 Methods

### 2.1 Data

The SMAP L-band (1.41 GHz) TB record used to derive the soil FT classification in this study was obtained from the SMAP radiometer twice-daily enhanced-resolution TB product (https://nsidc.org/data/nsidc-0738/versions/2), which is provided in a 9 km polar EASE-grid 2.0 projection (Brodzik et al., 2020). Here, the Scatterometer Image Reconstruction (rSIR) interpolation method originally developed for reconstructing images from raw scatterometer or radiometer data is used to provide spatially enhanced gridding of SMAP L1B radiometer half-orbit TB retrievals (Long et al., 2023). The rSIR interpolation exploits the increasing TB sampling from converging satellite polar orbital swaths and provides enhanced (~30 km) TB spatial resolution relative to the SMAP native sampling footprint (Long et al., 2023). The rSIR product is similar to other SMAP enhanced radiometer half-orbit TB products posted to the same 9 km grid projection, but derived using a different (Backus-Gilbert) spatial interpolation method optimized for global operations (Chaubell et al., 2020). Sampling from the sun-synchronous polar orbiting SMAP sensor occurs at approximately two-day intervals for land areas above 45°N, with consistent 6 p.m./a.m. local ascending/descending orbital overpass sampling times for the vertical (V) and horizontal (H) polarization TB retrievals. The SMAP rSIR period of record used for processing extended from March 31, 2015 to January 1, 2021. To construct complete daily records for each a.m. and p.m. TB time series, missing TB data between satellite swathes were gap-filled using a weighted average of the two most recent adjacent TB values within a five-day moving window, as:


(1) $$\begin{array}{l} T_{missing}=T_{prev}*\left(1-\frac{D_{prev}}{D_{prev}+D_{next}}\right)+T_{next}*\left(1-\frac{D_{next}}{D_{prev}+D_{next}}\right) \end{array}$$

where *T*_*missing*_ is the missing TB data being filled at a given location and time step, *T*_*prev*_ is the most recent retrieval before the missing data, *T*_*next*_ is the most recent retrieval after the missing data, *D*_*prev*_ is the number of days between *T*_*missing*_ and *T*_*prev*_, and *D*_*next*_ is the number of days between *T*_*missing*_ and *T*_*next*_. If no TB data was present within a given five-day window, the pixel was then masked out to prevent gaps from being filled with data too temporally distant.

AMSR2 TB data were obtained for the 18.7 GHz and 36.5 GHz channels, and V and H polarizations overlapping with the same period of record as that of SMAP. The Japan Aerospace Exploration Agency GCOM-W1 AMSR2 Level-3 gridded L1R TB data were obtained in a consistent 10 km resolution global EASE-grid format (Maeda et al., 2016). The AMSR2 TB record includes twice-daily coverage at higher latitudes owing to a relatively wide sensor swath and consistent 1:30 a.m./p.m. local sampling from the satellite sun-synchronous polar orbit. Missing AMSR2 TB data were gap-filled in the same manner as the SMAP data (above). The AMSR2 data were then reprojected to the same 9 km polar EASE-grid 2.0 format as the SMAP data using the Python LinearNDInterpolator program available from the SciPy software package. A distance weighted Barycentric interpolation method (Hofreither, 2021) was used to provide a smooth reprojection of the data by considering all nearby cells when calculating the values for each 9 km polar grid cell.

**Figure 1:**
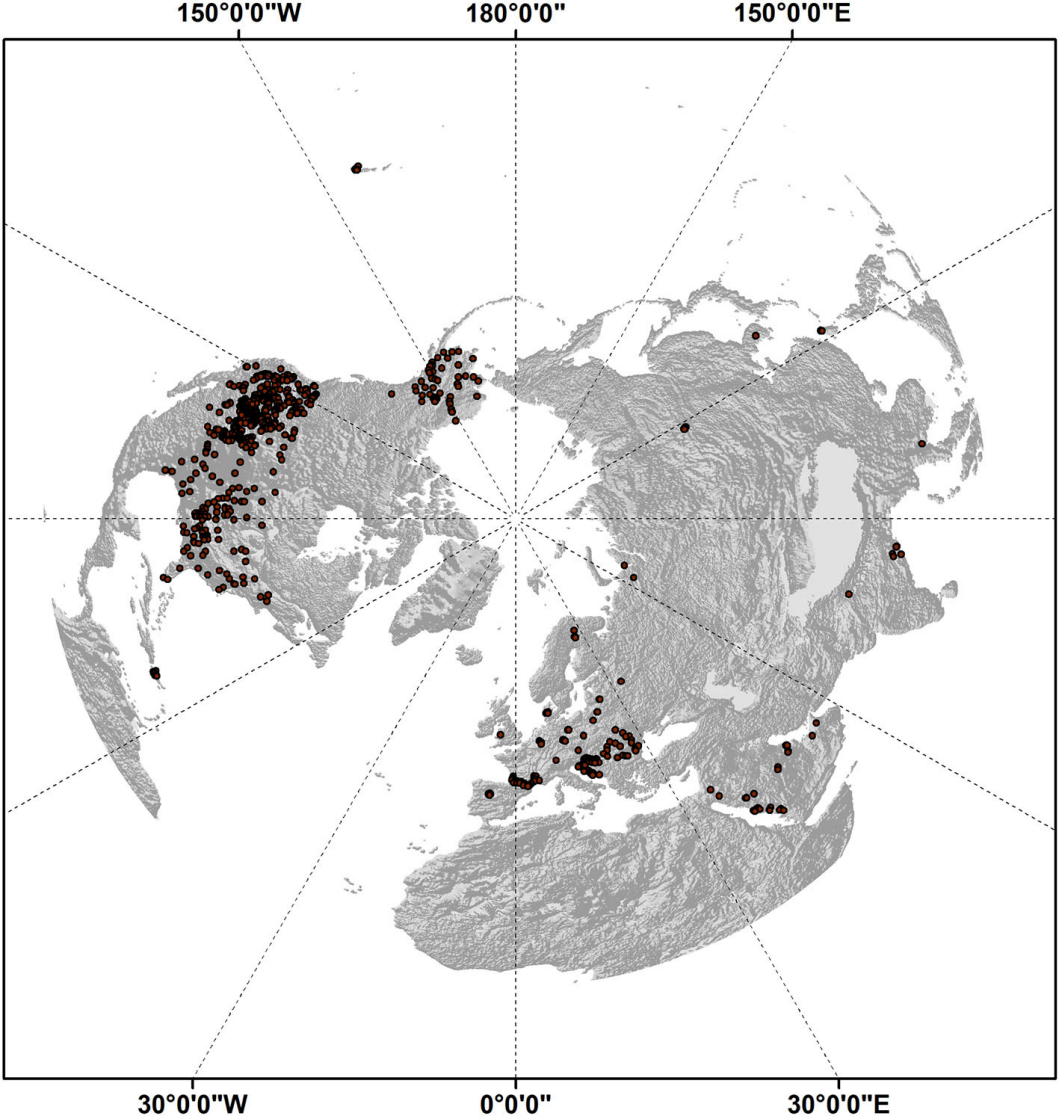
Shaded relief map showing the distribution of Northern Hemisphere weather station locations used in this study, which included *in situ* daily soil temperature measurements for the 2016–2020 period. A total of 804 stations are represented.

The above multi-frequency V and H polarized TB records from SMAP and AMSR2 provided the only dynamic daily inputs to the DL data model for the soil FT predictions. Additional model inputs included a static global digital elevation model (Danielson and Gesch, 2011) aggregated from the 1 km native resolution to the 9 km polar EASE-grid 2.0 projection. A global land cover map (Friedl et al., 2010) was used to identify and mask grid cells dominated by large water bodies, permanent ice and snow, and other non-soil areas from the model domain. Soil temperature data from *in situ* weather station measurements (Table 1) and gridded global meteorological reanalysis data were used for DL data model training and validation of the soil FT predictions. Near surface (0–5 cm depth) soil temperature measurements were obtained for approximately 800 Northern Hemisphere (NH) weather stations assembled from the Water and Climate Information System (USDA Natural Resources Conservation Service, 2017), International Soil Moisture Network (Dorigo et al., 2021), Global Terrestrial Network for Permafrost (GTN-P, 2015), and Global Learning and Observations to Benefit the Environment (GLOBE, 2021) networks. The distribution of stations used in this study is shown in Figure 1, although not all stations were active over the entire study period. For each station location, only the shallowest soil temperature readings (within 5 cm depth) were selected, and as close as possible to the SMAP 6 a.m./p.m. local sampling times. The location of each station measurement was assigned to the nearest 9 km grid cell. If multiple stations were assigned to the same grid cell, then the associated temperatures were spatially averaged to produce a single representative value for the cell at each time step. The a.m. and p.m. soil temperature measurements were then classified into frozen and non-frozen categories using a constant 0°C FT threshold.

**Table 1:** Descriptions of the *in-situ* soil temperature data sets.

**Dataset name**	**Description**	**References**	**Website**
Global terrestrial network for permafrost (GTN-P) Circumpolar active layer monitoring network (CALM) database	Ground temperatures along 1-ha and 1 km^2^ grids for Alaska and Canadian sites	GTN-P, 2015	https://gtnp.arcticportal.org/
Water and climate information system	Soil climate data are collected at automated Soil Climate Analysis Network (SCAN) sites throughout the U.S.	USDA Natural Resources Conservation Service, 2017	https://www.nrcs.usda.gov/
International soil moisture network	An international cooperation to establish and maintain a global *in-situ* soil moisture and soil temperature database	Dorigo et al., 2021	https://ismn.earth/en/dataviewer/
Global learning and observations to benefit the environment	Daily monitoring of the atmosphere along with soil temperature and moisture	Boger and Bagayoko, 2002	https://www.globe.gov/

The weather station soil temperature measurements reflect actual ground conditions useful for model validation, but the stations lack consistent sampling and are sparsely located. The actual number of station measurements also varies over time and may not be representative of the coarser model and satellite footprints, which can introduce uncertainty. To compensate for the limitations of the sparse station network, we also included gridded daily surface soil temperatures from ECMWF ERA5 global reanalysis data. ERA5 is a state-of-the-art model and data reanalysis product produced globally in a 30 km resolution and hourly time step (Hersbach et al., 2020). For this study, we used the ERA5 daily 6 a.m. and 6 p.m. Layer 1 (0–7 cm depth) soil temperatures. The ERA5 temperatures were reprojected to the 9 km polar EASE-grid 2.0 projection using the same method as the AMSR2 data. ERA5 temperatures were also converted to soil FT categorical values following the same procedure used for the *in situ* temperature processing. Because ERA5 is a model-based product rather than a direct measurement, we assume that the *in situ* soil temperature measurements at the station locations are more robust for validation, although the station measurements also have limitations, as noted above.

### 2.2 Model architecture and training

The DL data model we utilized for soil FT prediction employs a multi-layer convolutional neural network architecture called U-Net, originally developed for spatial image segmentation and pattern recognition (Ronneberger et al., 2015). The U-Net architecture consists of two parts: an encoder and a decoder. The encoder generally employs down-sampling to decrease the spatial dimension of the feature map and increase the number of feature channels. On the other hand, the decoder utilizes up-sampling to restore the spatial dimensions, and enrich feature details by merging the low-level and high-level feature maps across layers using concatenation operations (Ronneberger et al., 2015).

**Figure 2:**
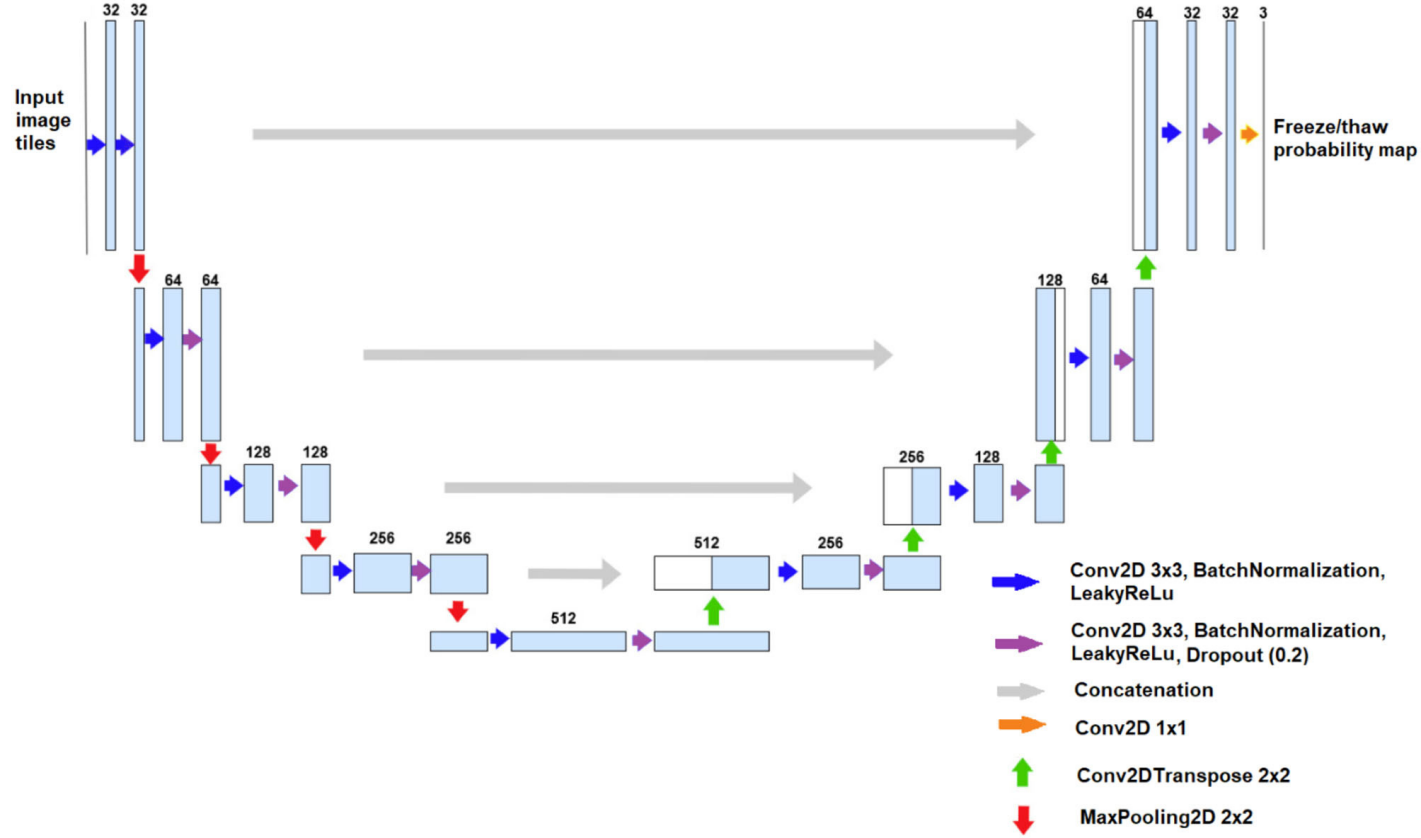
U-Net model architecture used in this study, where the blue boxes represent multi-channel feature maps with the number of channels labeled; the white boxes represent the copied feature maps, and the arrows denote different types of operations performed as detailed in the legend (Conv2D for 2D convolution operation, and 3 × 3, 2 × 2, and 1 × 1 for different kernel sizes).

For the U-Net architecture used in our soil FT prediction model (Figure 2), the encoder performs four down-sampling operations via maximum-pooling on convolutional blocks, each of which is defined by two sequences of a 2D convolution operation with 3 by 3 kernel size, followed by 2D batch normalization and leaky rectified linear units (ReLU) activation (Eckle and Schmidt-Hieber, 2019). The decoder performs four up-sampling operations via transposed convolution on the convolutional blocks and four concatenation operations performed through a skip connection. Finally, an output classification map is generated via 2D convolution with 3 by 3 kernel size to represent the probabilities of freeze and thaw states for each pixel at its original resolution. The primary difference between our model and the standard U-Net is the inclusion of spatial dropout layers at the end of each convolutional block after up-sampling or down-sampling operations, with a dropout rate of 20% (Tompson et al., 2015). Dropout is used alongside strong L2 regularization (Neyshabur et al., 2014) to prevent over-saturation of model weights, which is a particular concern due to the sparse station temperature data used for model training. This approach helps prevent overfitting in grid cells with station observations and anomalous differences in predictions relative to surrounding grid cells.

Model training and verification were conducted using soil temperature based FT observations from data years 2017, 2018, and 2019. The model soil FT predictions were also validated using FT observations from years 2016 to 2020 that were excluded from the model training. After each epoch, the resulting models were evaluated using a combination of data from different training years, while the best performing model was selected for final production. Both Binary Cross Entropy (BCE) and local-variational cost functions (Ruby et al., 2020) were used to maximize the performance of the model FT predictions against the observational FT reference data used for model training. BCE is a standard for binary classification problems such as FT predictions. It takes the model's predicted probabilities for frozen or thawed conditions, compares them to the FT reference defined from the observation training data, and then penalizes the neural network based on the distance between the model predicted and expected values. To obtain a probability distribution over FT classes for each pixel, an activation function is applied to the U-Net. The function normalizes the raw network outputs (logits) into a probability distribution. The output of the model is passed through a sigmoid activation function to produce probabilities for each class channel. A probability map is produced by normalization along each channel dimension and then selecting the thawed class channel as the final product output. The probability output of the FT classification can be interpreted as a measure of the model uncertainty about the classification decision. It indicates the model confidence or uncertainty in the estimated surface soil FT conditions given the input satellite observations and other ancillary data. This procedure pushes the model predictions to be closer to 0 (indicating 0% likelihood of thawed condition, signifying frozen) or 1 (100% likelihood of thawed condition) when there is greater confidence in the prediction and closer to 0.5 (representing an equal chance of being classified as frozen or thawed) when the FT status is uncertain. Therefore, in addition to the discrete binary (0 or 1) FT classification, the resulting model provides a continuous variable estimate, ranging between 0 and 1, of the probability of frozen or thawed conditions.

The local-variation loss function spatially smooths the model predictions by penalizing differing FT predictions between neighboring grid cells. Local-variation loss is calculated by taking the L_1_-norm of a grid cell and its neighbors vertically and horizontally, and then summing the vertical and horizontal components (Luo et al., 2016). This procedure was used to avoid model overfitting for grid cells containing sparse *in situ* weather station data in addition to the more continuous training data provided from ERA5 reanalysis temperatures, which can lead to these cells behaving differently from neighboring grid cells lacking weather station data. A general assumption of this approach is that a given grid cell will show similar soil FT behavior as neighboring grid cells except in areas with large FT heterogeneity, which commonly occurs in complex mountain terrain. A drawback of the local-variation loss function is that it can impose excessive smoothing of heterogeneous FT patterns by penalizing predictions that differ from surrounding grid cells, which may degrade model performance particularly in mountain regions. To counteract potentially excessive smoothing, the local-variation loss function was assigned a relatively low unitless weighting factor (0.1) to lessen the overall smoothing effect. Model accuracy was evaluated using the Matthews Correlation Coefficient (MCC). The MCC accounts for all four factors of the confusion matrix relative to the respective number of positive and negative elements in the data, allowing it to work even if there is a large class imbalance (Boughorbel et al., 2017). The MCC was calculated as follows:


(2) $$\begin{array}{l} MCC=\frac{TP*TN-FP*FN}{\sqrt{\left(TP+FP\right)\left(TP+FN\right)\left(TN+FP\right)\left(TN+FN\right)}} \end{array}$$

where TP is the number of true positives, TN is the number of true negatives, FP is the number of false positives, and FN is the number of false negatives. The MCC values range from 1 for perfectly correlated predictions to −1 for perfectly uncorrelated predictions. At the end of each training epoch, the model was saved whenever a higher MCC score was achieved in relation to the validation data. We also calculated the F1 score as follows, which combines precision and recall into a single score for assessing the overall performance of the U-Net:


(3) $$\begin{array}{l} F1=\frac{2TP}{2TP+FP+FN} \end{array}$$

We trained five different DL data models using the same U-Net architecture and training data, but with different combinations of geospatial inputs to predict daily soil FT conditions. Each model was developed using a different set of satellite TB inputs designed to clarify the relative utility of the different sensors and microwave frequencies on the resulting model performance in relation to soil FT observations from ERA5 reanalysis and *in situ* weather station measurements. The five different models were developed using different combinations of TB inputs as follows: SMAP 1.4 GHz (model 1); AMSR2 18.7 GHz (model 2); AMSR2 36.5 GHz (model 3); AMSR2 18.7 and 36.5 GHz (model 4); and combined SMAP 1.4 GHz, AMSR2 18.7 and 36.5 GHz inputs (model 5). All other ancillary geospatial inputs were consistent across models. Differences in the resulting model performances in estimating soil FT were used to gage the relative value and impact of the different TB frequencies. The five models were developed and tested for local morning (6 a.m.) conditions aligned with the SMAP descending orbit TB inputs, and spanning all NH land areas. The best performing model TB channels from the above comparison were then used to train a similar model for predicting daily soil FT conditions for the local evening (6 p.m.) period corresponding with the SMAP ascending orbital overpass time. Here, both the SMAP and AMSR a.m. (p.m.) TB records, which represent different satellite overpass times, were used as model inputs to estimate soil FT conditions in the morning (evening); whereas, the model training was conducted using soil temperature observations selected for local 6 a.m. (6 p.m.) conditions corresponding with the SMAP sampling times. Two different metrics were used for evaluating the resulting model soil FT performance. For the model daily binary FT outputs, the Mean Percent Accuracy (MPA) metric was used to quantify the percentage of correct predictions to the overall number of predictions made. For the model probabilistic (i.e., probability of thawed conditions) outputs, the Brier score metric was used to assess model performance. The Brier score ranges between 0 and 1, where a lower score indicates greater accuracy. The Brier score is similar to the Mean Squared Error in measuring how close a probabilistic prediction is to a true classification and is calculated as:


(4) $$\begin{array}{l} B=\frac{1}{N}\overset{N}{\underset{i=1}{\sum }}{\left(P_{i}-T_{i}\right)}^{2} \end{array}$$

Where *N* is the number of predictions, *P*_*i*_ is the predicted probability at time *i*, *T*_*i*_ is the true frozen or thawed (binary 0 or 1) value at time *i*.

## 3 Results

### 3.1 Identifying the best performing DL data model

Table 2 shows the performance of the five DL data models in relation to ERA5 and weather station temperature based soil FT observations for the entire study period (2016–2020). Overall, the single-frequency TB models performed worse than the multi-frequency models. The model using only SMAP TB inputs showed the lowest performance and accuracy against the ERA5 reference but had the highest weather station accuracy of all single frequency models. All of the models showed better performance against the weather station observations than ERA5, indicating potential value of the satellite based soil FT classification records to inform global land model predictions. The dual-channel AMSR model performed better than the single frequency models, but the combined AMSR+SMAP (18.7, 36.5, 1.4 GHz) model showed the best performance of all models, with 3.5 and 2% improvements over the dual channel AMSR model in relation to the respective ERA5 and weather station observations. The combined AMSR + SMAP model, developed for the p.m. period, showed very similar performance to the a.m. results, and also showed consistently greater accuracy than all other models tested. These results imply that the SMAP L-band TB observations have a different sensitivity to soil FT processes than the higher frequency AMSR2 TB observations. Moreover, the combined multi-frequency observations provide complementary information enabling the highest soil FT performance.

**Table 2:** Overall MPA (%) and Brier scores for the 2016–2020 study period estimated between each DL data model and ERA5 and weather station derived soil FT observations.

	**ERA**		**Weather stations**	

	**MPA**	**Brier score**	**MPA**	**Brier score**
ERA5	100	N/A	85.0	N/A
SMAP	84.5	0.1038	89.4	0.0883
AMSR18	89.5	0.07661	89.3	0.0880
AMSR36	87.9	0.0851	89.2	0.0892
AMSR18 + 36	90.0	0.0773	90.0	0.0890
AMSR + SMAP (a.m.)	**92.9**	**0.0508**	91.0	**0.0769**
AMSR + SMAP (p.m.)	92.4	0.0581	**91.1**	0.0779

The ERA5 record is compared against itself and the weather station observations as a baseline for gaging model performance. The different DL data models were derived using SMAP 1.4 GHz (SMAP), AMSR2 18.7 GHz (AMSR18), AMSR2 36.5 GHz (AMSR36), AMSR2 18.5 and 36.5 GHz (AMSR18 + 36), and combined AMSR2 and SMAP (AMSR + SMAP) TB inputs for morning (6 a.m.) conditions. The AMSR + SMAP results include both morning and evening (6 a.m./p.m.) periods. The best performing metrics are highlighted (in bold).

### 3.2 Spatial and temporal consistency in estimated soil FT characteristics

**Figure 3:**
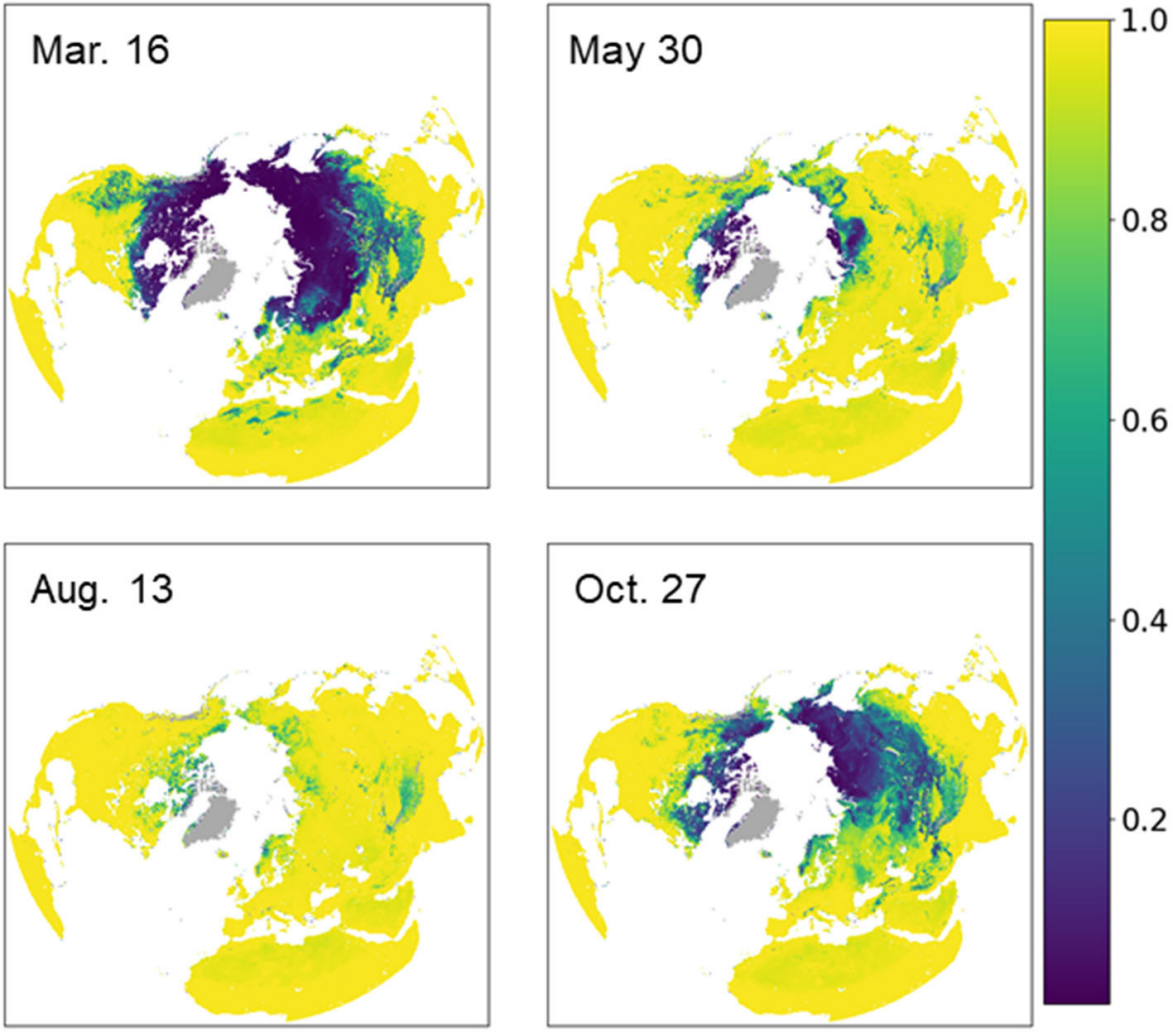
The estimated probability of thawed soil conditions for four selected days over the 2016 seasonal cycle in the Northern Hemisphere; permanent ice/snow and other non-soil areas outside of the model domain are shown in gray. The probability of thawed conditions ranges from low (0) to high (1) and is lower at higher latitudes and altitudes during early spring (March) and late fall (October) periods; in contrast, the probability of thawed conditions is much greater during mid-summer (August).

Daily soil FT conditions were estimated over the 2016–2020 study period using the best performing DL data models identified from the above analysis, which incorporated multi-frequency TB inputs from overlapping AMSR2 and SMAP sensor records. Different DL data models were used for estimating morning (6 a.m.) and evening (6 p.m.) FT conditions from the collocated orbital TB retrievals. The distribution of the estimated probability of thawed (and frozen) soil conditions is shown in Figure 3 for four selected days spanning the NH annual cycle. The probability of thawed conditions ranges from 0 to 1 and is lower and less extensive at higher latitudes and altitudes during early spring (March) and late fall (October) periods. In contrast, the probability of thawed conditions is much greater and more extensive during mid-summer (August). The results capture the large characteristic variation in FT conditions across the domain, where higher latitudes and alpine elevations show a generally higher probability of frozen conditions. The probability of thawed soil conditions is greater at lower latitudes and elevations, and along coastal margins where soil conditions have greater exposure to moderating temperatures and more transient frost events. Frozen (or thawed) conditions may also occupy a smaller (larger) proportion of a given grid cell in these areas, manifesting as a lower (higher) estimated probability. Regions with complex terrain, including the Qinghai-Tibetan Plateau and North American Rocky Mountain regions, also show greater heterogeneity in FT conditions due to greater terrain, land cover, and microclimate variability. The model confidence and estimated probability of frozen or thawed conditions is also generally lower in these areas due to the greater FT heterogeneity relative to the coarse model grid.

**Figure 4:**
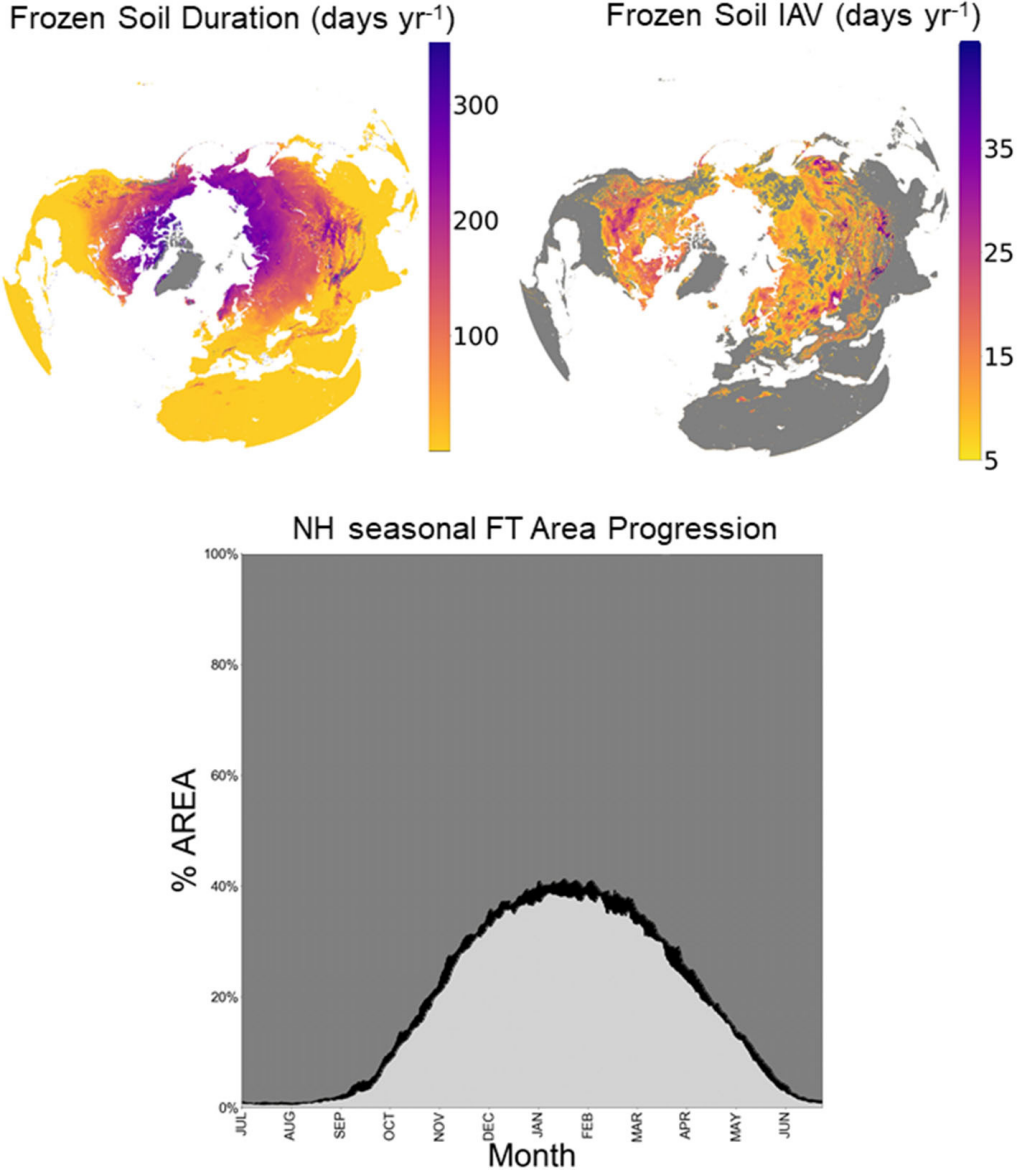
Estimated mean annual duration (days) in frozen soil conditions from 2015 to 2020, where large water bodies, permanent ice and other masked areas are shown in white and gray **(top left)**. The associated interannual variation (IAV = ±1 temporal SD, days yr^−1^) in the frozen season is presented, where areas with low variability (<±5 days yr^−1^) or infrequent frost events are shown in gray **(top right)**. The seasonal progression in the estimated mean proportional (%) frozen soil extent over the NH domain, excluding non-soil areas, is also shown **(bottom)** as derived from the a.m. soil FT record, where the mean frozen area is shaded light gray, and the IAV in frozen area is shown in black; the proportional non-frozen area is represented by the dark gray shading in the same plot.

The estimated mean annual duration (days) and interannual variation (±1 SD, days yr^−1^) in frozen soil conditions over the NH domain is shown in Figure 4. As expected, frozen soils persist over a greater portion of the year at higher latitudes and elevations. The frozen season spans the majority of the annual cycle across the Arctic, including the Alaskan North Slope, Canadian Northwest Territories, northern Scandinavia, and northeastern Eurasia. In contrast, the frozen season covers less than half of the year in the boreal regions of central and southwestern Alaska, Canada, and Eurasia. Interannual variability (IAV) in the frozen season is generally greater at lower latitudes and altitudes, and along the boundaries of different major air masses and climate regimes. For example, the northern Great Plains region of North America shows relatively large frozen season IAV of up to 3 weeks or more, consistent with frequent shifts in the boundaries between colder polar and continental interior air masses, and warmer maritime and temperate air masses that occur during seasonal transitions. Significant frozen soil conditions also occur at lower latitudes and are mainly concentrated in the Qinghai-Tibet Plateau and other high elevation areas. More sporadic frozen soil conditions are also located in mountainous areas in Northern Africa, Southern Europe, and North America.

The mean seasonal progression in NH frozen soil extent derived from the a.m. FT classification record is also plotted in Figure 4. Here, frozen soil conditions range from respective annual maximum to minimum extents between January and July. The fall (October–December) and spring (March–May) seasonal transitions coincide with rapid changes in frozen area. The summer minimum extent of frozen soil conditions is less than the area of continuous permafrost cover across the Arctic (e.g., Zhang et al., 2008) because the FT classification is more representative of the surface of the seasonally thawed soil, or active layer, overlying the deeper permafrost layer. The IAV in the seasonal maximum frozen area extent averages ±0.88 percent of the classified NH land area, excluding permanent ice and snow, and other non-soil areas. The IAV is also generally larger (±1.03%) during the spring and fall transition periods. The seasonal progression of the non-frozen soil area is also represented by the dark gray color in the same plot. The spatial extent of thawed soils exceeds the frozen area in all months of the year over the Northern Hemisphere. Non-frozen soil conditions extend over nearly all of the NH domain in mid-summer (Jul-Aug) but contract to cover a minimum of approximately 60% of the NH in late winter (Jan-Feb), and mainly in the lower latitudes where frost events are infrequent.

**Figure 5:**
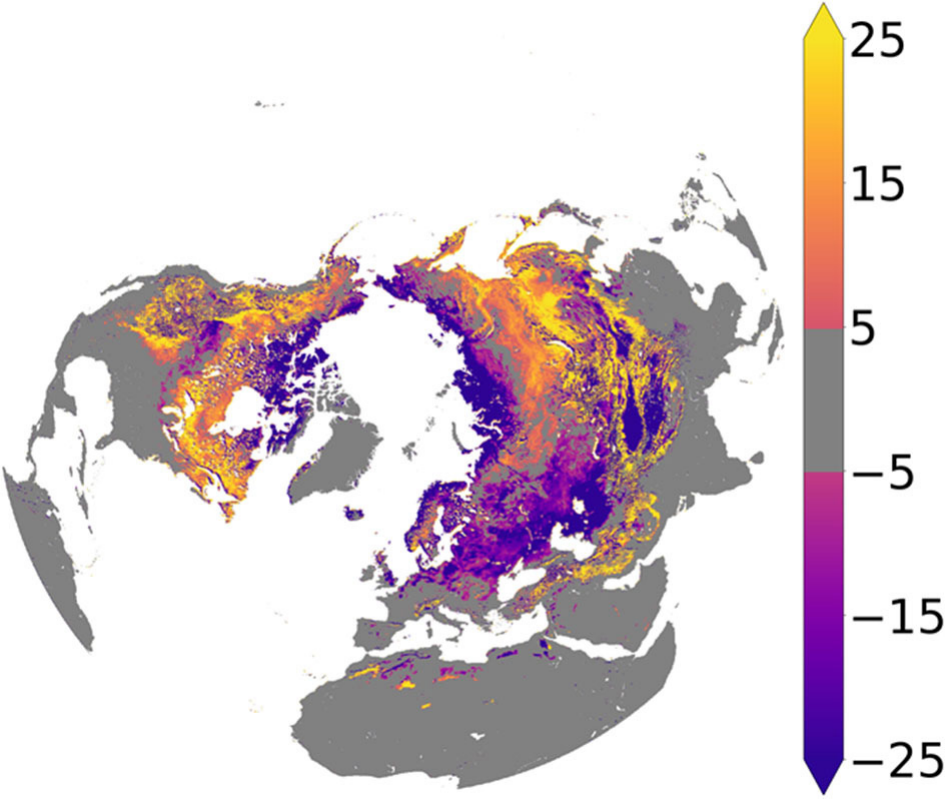
Difference in estimated mean annual frozen period (days) derived from the 6 a.m. and 6 p.m. soil FT records (a.m.-p.m.).

The spatial pattern of the difference in the estimated mean annual frozen period derived from the soil FT classification records for the morning (6 a.m.) and evening (6 p.m.) periods is presented in Figure 5. The mean absolute difference (days yr^−1^) in the frozen soil season duration estimated from the a.m. and p.m. records is 24 (SD ± 20) days, which is small compared to the frozen season length. The a.m. classification shows a generally longer frozen season than the p.m. classification over more than 77% of the domain, consistent with the characteristic diurnal cycles of mid-day thermal maximums and nighttime minimums. However, a reversed pattern of longer p.m. than a.m. frozen seasons occurs across much of the Arctic, northern Europe, western Eurasia, and the Qinghai-Tibetan Plateau. These regions have generally low statured shrub and grassland vegetation, where the daily FT cycle under minimal snow cover may be reversed due to insulating nighttime cloud cover and associated seasonal and diurnal changes in solar radiation loading and surface energy exchange (Ross et al., 1996; Lakshmi et al., 2001). The reversed diurnal pattern may also be an artifact of greater regional model uncertainty due to the abundance of small water bodies in the high northern latitudes and their contaminating influence on the TB observations over land (Du et al., 2016, 2023; Kim et al., 2019).

**Figure 6:**
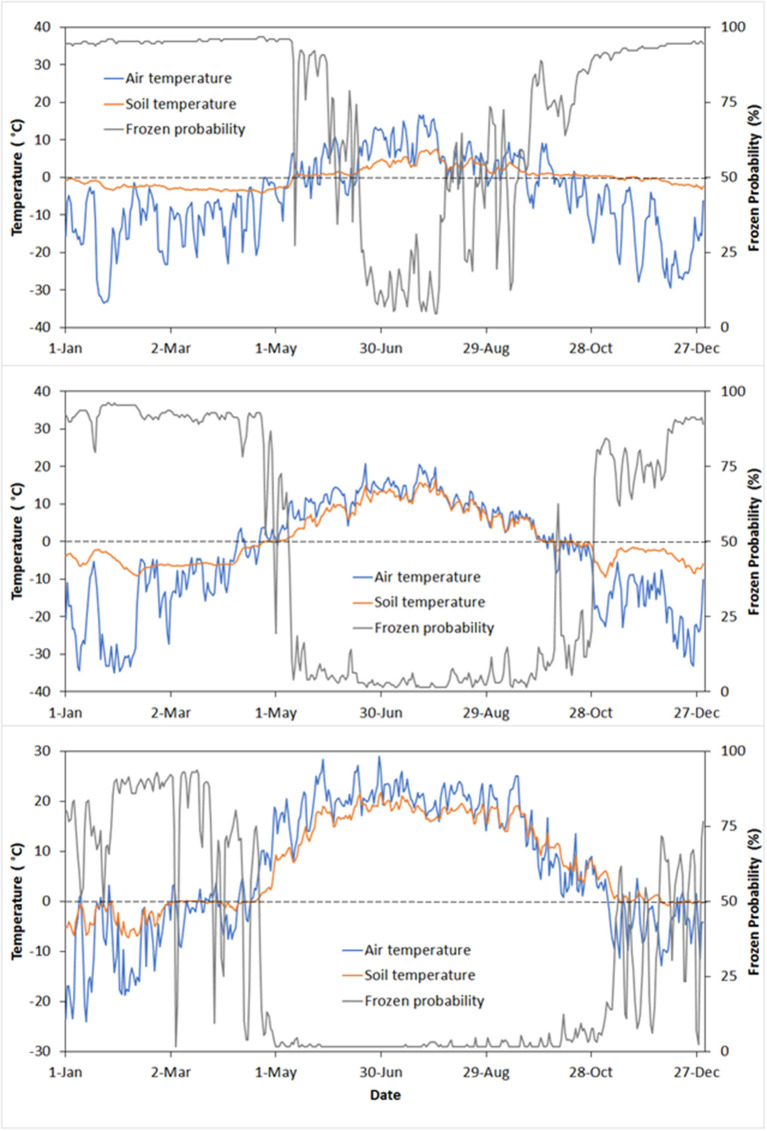
Estimated daily probability (%) of frozen soil conditions at 6 a.m. local time over selected year 2018 for three Ameriflux site locations spanning a latitudinal, climate, and vegetation gradient. The **top plot** represents Arctic tundra (Imnavait Creek, AK, 69.6063N, −149.3041W); the **middle plot** is boreal evergreen needleleaf forest (Fairbanks AK, 64.8663N, −147.8555W); and the **bottom plot** is northern temperate grassland (Rosemount MN, 44.6781, −93.0723W). The satellite based frozen soil probabilities represent the 9 km resolution grid cell overlying each site location, relative to corresponding mean daily air and surface (2 cm depth) soil temperatures from the local site measurements. The horizontal dashed line depicts the 0°C threshold between pure liquid water and ice.

The seasonal progression in the estimated probability of frozen soil conditions is shown with collocated *in situ* daily air and soil temperature measurements at selected tundra, boreal forest and grassland monitoring sites spanning a latitudinal climate and vegetation gradient (Figure 6). The selected sites are part of the AmeriFlux tower network (Novick et al., 2018) and are independent from the model training and validation. The estimated frozen soil conditions closely track the soil temperature seasonal progression indicated from the site measurements, ranging between annual maximum and minimum probabilities from late winter to summer. Surface air temperatures at the sites show greater variability and a larger dynamic range that is both colder in winter and warmer in summer than the observed soil temperatures. The greater soil temperature stability is consistent with the larger soil heat capacity than air and the effects of thermal buffering from insulating winter snow cover at the sites. The probability of frozen soil conditions is nearly 100% in late winter and early spring prior to seasonal thawing and corresponding with frozen soil temperature measurements that are near seasonal lows. Relatively rapid warming and thawing of soils during the spring onset coincides with a large drop in the estimated frozen soil probability. The spring thaw transition begins earlier and includes more transient thaw and refreeze events at the temperate grassland site. In contrast, the spring thaw transition occurs later at the boreal and tundra sites. The estimated probability of frozen soil conditions is higher and more temporally stable in late winter and early spring at the Arctic tundra site, consistent with the colder and more stable conditions preceding spring onset. In contrast, the temperate grassland site shows a lower and more variable frozen soil probability coincident with soil temperature measurements that persist near the 0°C FT threshold.

The frost-free season indicated by the estimated low probability in frozen soil conditions spans the summer months and has the shortest duration at the Arctic tundra site, is intermediate at the boreal forest site, and has the longest duration at the temperate grassland site. The fall onset in frozen soil conditions indicated from the temperature measurements coincides with a persistent increase in estimated frozen soil probability from the summer minimum, which begins first at the tundra site, extending to the boreal site, and occurring last at the grassland site. The probability of frozen soil conditions shows a general increasing trend into the winter season coinciding with the continued cooling of soil and air temperatures after initial freezing. The soils persist near 0°C longer than the overlying air temperatures during the fall freeze transition, which may reflect the influence of thermal buffering from latent heat release during soil ice formation and the arrival of seasonal snow cover (Kane et al., 2001; Zhang et al., 2018). The grassland site shows a relatively low, but temporally varying probability of frozen soil conditions during the fall transition coinciding with a persistence of soil temperatures near 0°C and transient thaw and refreeze events extending into the winter season. The tundra site shows a relatively early, but variable increase in frozen soil conditions in mid-August, which precedes the arrival of a more stable frozen season by October. Frozen soil probabilities during this period, while elevated relative to summer lows, remain low (near 50%) compared with the winter months and coincide with soil temperature measurements above 0°C, but with transient frost events indicated from the air temperature measurements. The tundra site also shows greater variability in frozen soil probabilities during the summer season; the larger variability and uncertainty in the model predictions reflects the topographic complexity of the surrounding landscape at this site, which is located in the foothills of the Alaskan Brooks Range. Here, the greater microclimate and FT spatial heterogeneity encompassed by the coarse (9 km resolution) model grid cell may be different from local site conditions represented from a single weather station.

### 3.3 Validation against ERA5 and weather station observations

**Figure 7:**
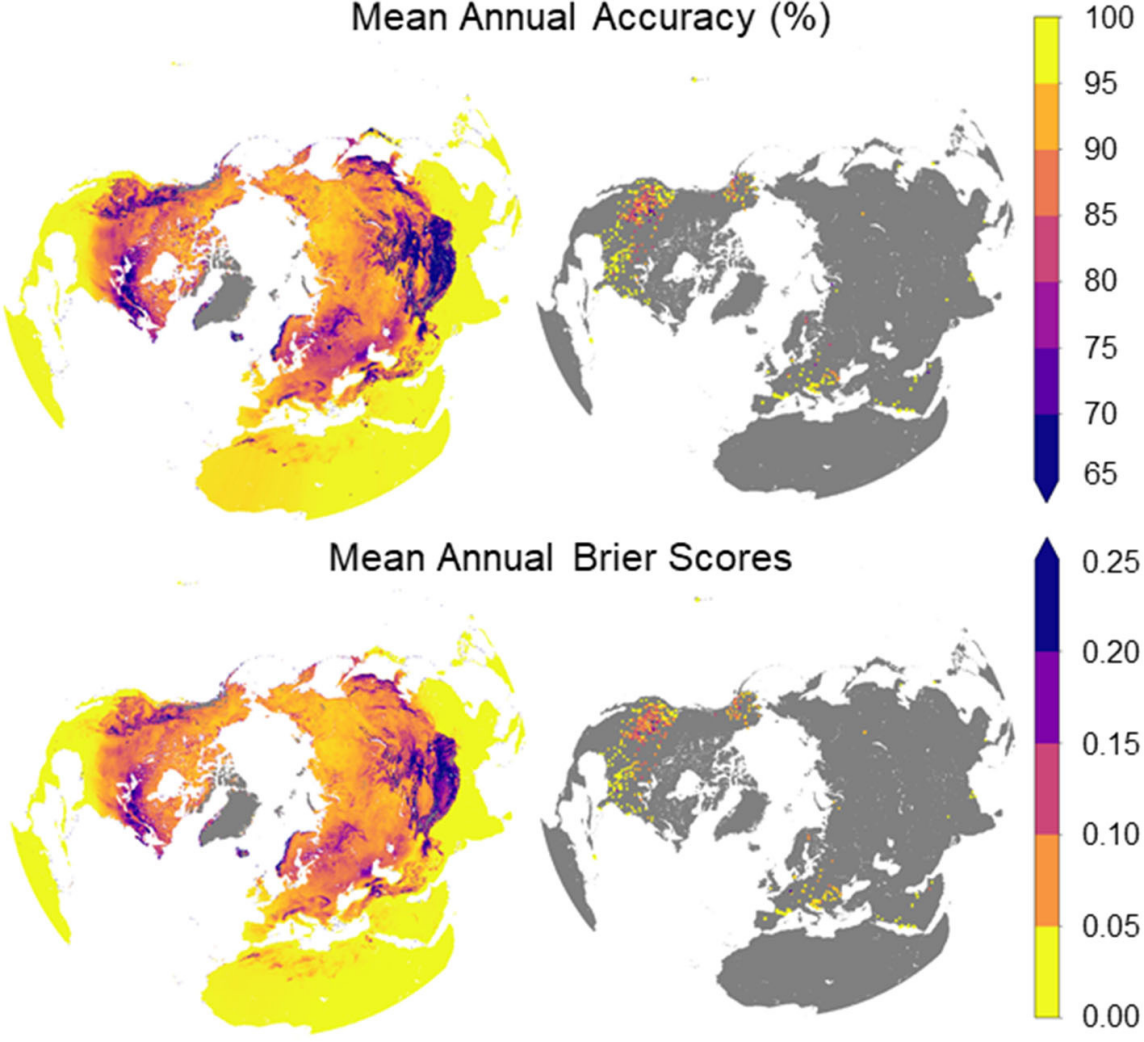
Pattern of estimated mean percent accuracy of DL based soil FT predictions for the study period (2016–2020) indicated from ERA5 **(top left)** and NH weather station soil temperature records **(top right)**; Brier Scores indicating similar probabilistic soil FT accuracy are also shown against ERA5 **(lower left)** and weather station network **(lower right)** observations for the same period. Here, warmer colors denote better model performance.

The spatial performance of the DL soil FT classification in relation to the FT reference data from ERA5 and the weather station network (WS) is shown in Figure 7. The estimated DL performance pattern is similar between the MPA and Brier score metrics, although a lower Brier score indicates better performance. The estimated DL performance is also similar against both ERA5 and the weather station observations. As expected, the highest accuracy occurs in lower latitude regions where FT events are a rare occurrence. Similar high accuracy levels occur over the polar high latitudes where the frozen season is relatively stable and occupies a larger portion of the annual cycle. The performance is lower, but still favorable over northern temperate and boreal regions, which have longer FT transitional seasons. Lower performance is indicated over portions of central and eastern North America, with slightly lower accuracy indicated from ERA5 than the weather station observations in these areas. These areas are characterized by dynamic weather and seasonal climate variations affecting snowmelt and soil FT processes that are difficult to capture from global climate models (Morcrette et al., 2018; Dutra et al., 2021). Relatively low accuracy also occurs over high elevation areas and complex terrain, including the Rocky Mountains and Qinghai-Tibetan Plateau. The lower apparent skill in these regions is consistent with the greater spatial complexity in FT conditions, which may be below the effective resolution of both the soil FT classification and the sparse station observations, and the global reanalysis data used for DL training and validation (Liu et al., 2020).

The mean annual and seasonal performance of the DL estimated soil FT classification record is summarized in Table 3. Overall, the MPA of the DL results in relation to the ERA5 (and WS) reference data ranged from 90.1 (83.1) percent in winter to 97.9 (98.9) percent in summer, and 91.5 (91.2) percent during the spring and fall transition seasons. Differences in model accuracy between a.m. and p.m. conditions were minimal and within 1.6 (1.0) percent of each other across all seasons. The model accuracy was also mostly similar against both ERA5 and WS observations, although the apparent product performance was approximately 8% lower against the WS observations in winter compared to the ERA5 reference data. The mean annual accuracy of the model soil FT classification was 92.7 (91.0) percent over the entire NH domain. The model accuracy was similar among different land covers, including forest (91.1%) and non-forest (90.6%) types. The model accuracy was slightly lower, but still favorable against the ERA5 (87.8%) and WS (90.5%) observations when only grid cells with a significant number (>5 days yr^−1^) of FT events were considered. The apparent accuracy of the DL soil FT product is higher than reported (accuracy 80% to 90.3%) from other similar satellite FT records derived from SMAP (Derksen et al., 2017) or AMSR (Kim et al., 2017) TB records. Additional assessment also confirms high consistency between the DL and ERA5 annual results with F1 score 0.905. The model accuracy is also higher than the apparent ERA5 accuracy when both are compared to the WS soil FT data. These results indicate that the product provides a relatively high level of accuracy and consistency between a.m. and p.m. conditions, across seasons, and over the NH domain. The product also provides better soil FT accuracy than an advanced global model reanalysis system against regional WS network observations for the period examined.

**Table 3:** Mean annual and seasonal percent accuracy and Brier scores (shown in parentheses) between model estimated soil FT and ERA5 and Weather Station (WS) observations.

	**Annual**		**Spring (MAM)**		**Summer (JJA)**		**Fall (SON)**		**Winter (DJF)**	
	**ERA5**	**WS**	**ERA5**	**WS**	**ERA5**	**WS**	**ERA5**	**WS**	**ERA5**	**WS**
ERA	100	86.1	100	83.0	100	94.2	100	89.4	100	73.3
U-Net a.m.	93.0 (0.05)	90.9 (0.06)	92.7 (0.05)	90.2 (0.07)	98.6 (0.01)	99.2 (0.01)	91.2 (0.06)	92.2 (0.06)	89.3 (0.08)	82.6 (0.12)
U-Net p.m.	92.3 (0.06)	91.1 (0.07)	91.3 (0.06)	90.5 (0.07)	97.2 (0.03)	98.7 (0.01)	90.3 (0.07)	91.9 (0.06)	90.9 (0.07)	83.6 (0.12)

The U-Net (model 5) performance is summarized for a.m. and p.m. conditions. Soil FT estimates from ERA5 are also assessed against the *in situ* WS observations for reference.

## 4 Summary and conclusion

We developed a continuous daily classification record of surface (0–5 cm depth) soil freeze-thaw dynamics spanning all Northern Hemisphere land areas and informed from satellite multifrequency TB observations from SMAP and AMSR2 as key model drivers. A deep learning (DL) method employing a novel U-Net neural network architecture and trained on integrated soil temperature observations from ERA5 global reanalysis and Northern Hemisphere weather stations was used to estimate twice-daily (6 a.m./p.m. local time) soil FT conditions. Unlike other satellite-based FT records that commonly represent a bulk landscape FT retrieval, the DL results are specific to FT conditions in the surface soil layer by being trained specifically on soil temperature observations and effectively exploiting TB observations with different but complimentary sensitivity to soil conditions and other landscape FT elements.

A comparison of different DL data models developed using both single frequency and multifrequency TB inputs from AMSR2 and SMAP showed the best performance and accuracy was achieved by combining AMSR + SMAP inputs. The DL performance was similar between morning (a.m.) and evening (p.m.) periods, while the combined multi-frequency TB observations produced the highest soil FT accuracy and consistency. These results indicate that the SMAP L-band (1.4 GHz) TB observations provide additional value over higher frequency (18.7, 36.5 GHz) measurements from AMSR2 for the soil FT predictions. Moreover, all of the resulting DL predictions showed better accuracy and performance against the weather station observations than ERA5, indicating potential value of the satellite-based soil FT classification records to inform earth system model predictions.

The resulting daily soil FT classification record is posted to a 9 km Northern Hemisphere polar EASE-Grid projection consistent with the SMAP rSIR spatially enhanced TB inputs (Brodzik et al., 2020). The product includes a simple binary FT classification similar to other established satellite microwave FT classification records (e.g., Derksen et al., 2017; Kim et al., 2017); however, the product also includes a continuous variable estimate of the probability of frozen or thawed soil conditions, which may be more suitable for some applications, including data assimilation (Farhadi et al., 2015). The resulting soil FT classification effectively distinguishes soil from other landscape elements, which may enable better precision and understanding of soil FT controls on other biophysical processes, including soil decomposition and greenhouse gas emissions (Kurganova et al., 2007), surface runoff (Wang et al., 2009), soil erosion and permafrost stability (Guo et al., 2018; Zhang et al., 2021). The data record contains soil FT predictions for local morning (6 a.m.) and evening (6 p.m.) conditions extending from data years 2016 through 2020. Potential continuity of the data record is enabled from ongoing operations of the NASA SMAP and JAXA AMSR2 missions. However, product performance may degrade as the classification record extends further away from the initial model training period, which may require periodic model retraining involving a progressively longer data record to maintain a consistent high level of performance.

The soil FT classification record developed from this study shows relatively high accuracy and stable performance over the NH domain and across seasons. The estimated accuracy against soil FT observations from ERA5 reanalysis and the regional WS network exceeded 90% for both a.m. and p.m. predictions. The seasonal variation in product accuracy was small (i.e., within 8%), indicating stable model performance. The product accuracy is also favorable and largely consistent across different land cover types, which differs from other satellite microwave FT retrievals that have shown degraded performance in forests (Kim et al., 2019; Walker et al., 2022). Overall, these results indicate a high level of accuracy and consistent performance relative to other established FT products. However, the model was trained on FT estimates derived from soil temperature observations using a static 0°C threshold to distinguish between frozen and thawed conditions, whereas the actual freezing point of soil water may occur at lower temperatures depending on the type and concentration of dissolved solutes (e.g., Pardo et al., 2020). The product also shows lower accuracies over complex mountain regions such as the Rocky Mountains and Qinghai-Tibetan Plateau. The lower accuracy in these regions partially reflects the relatively coarse (~30 km) resolution of the satellite TB inputs and ERA5 temperatures used for model training and validation. The available WS network also used for model training and validation is particularly sparse in the high northern latitudes and likely fails to capture the large microclimate and FT spatial heterogeneity in these regions. The use of local variation loss to prevent model overfitting when attempting to blend the relatively sparse weather station observations with the full ERA5 coverage may also produce excessive smoothing of temperatures in regions with complex terrain, leading to a loss of detail in the FT predictions. While the sparse distribution of available weather stations limits the model performance, particularly over complex terrain, continuing performance and spatial resolution enhancements in the reanalysis data used for model training may enable additional gains in model accuracy. For example, the latest generation ERA5-Land reanalysis provides enhanced (9 km) spatial gridding (Muñoz-Sabater et al., 2021) that may improve model training over complex terrain and land cover areas. The model performance may also benefit from the use of other available land parameters as model inputs, including satellite observational records of snow cover extent, soil moisture, and land surface temperature.

The U-Net architecture adapted for this study leverages the relative strengths of this method for image segmentation tasks. The method used in this study is similar to the original U-Net architecture, except for the addition of dropout layers. Other U-Net variations may further enhance model performance. Other network architectures, such as transformers, offer particular strengths in image recognition (Dosovitskiy et al., 2020) and segmentation (Chen et al., 2021; Strudel et al., 2021). These alternate architectures may allow for different training methods that avoid limitations from local variation loss and the network to generalize more efficiently.

## Data availability statement

The datasets presented in this study can be found in online repositories. The names of the repository/repositories and accession number(s) can be found at: http://files.ntsg.umt.edu/data/NH_SoilFT/DAILY_GEOTIFF/.

## Author contributions

KD, JK, and JD designed the study, conducted the analysis, and authored the paper. FB and JJ contributed to the U-Net design and related methods development. MM, AC, MR, and YK contributed to editing and writing of the final paper. All authors contributed to the article and approved the submitted version.
